# Supplementary material for: Spatial genomics maps the structure, nature and evolution of cancer clones
Source: Nature. 2022 Nov 9;611(7936):594–602. doi: 10.1038/s41586-022-05425-2 (PMC9668746; doi:10.1038/s41586-022-05425-2)
Supplement: Supplementary file 1 — This file contains Supplementary Methods; Supplementary Discussion Notes; Statistics and Reproducibility and Supplementary References. [file 41586_2022_5425_MOESM1_ESM.pdf]

nature portfolio

https://doi.org/10.1038/s41586-022-05425-2

Supplementary information

Spatial genomics maps the structure, nature and evolution of cancer clones

In the format provided by the authors and unedited

Spatial genomics maps the structure, nature and evolution of cancer clones

Artem Lomakin[1,2,3]†, Jessica Svedlund[4]†, Carina Strell[4,5], Milana Gataric[1,2], Artem Shmatko[3], Gleb Rukhovich[2,3], Jun Sung Park[1,2,3], Young Seok Ju[6], Stefan Dentro[1,2,3], Vitalii Kleshchevnikov[2], Vasyl Vaskivskyi[2], Tong Li[2], Omer Ali Bayraktar[2], Sarah Pinder[7,8], Andrea L Richardson[9], Sandro Santagata[10,11,12], Peter J Campbell[2], Hege Russnes[13,14], Moritz Gerstung[1,3]*, Mats Nilsson[4]*, Lucy R Yates[2]*

Table of Contents

Statistics and Reproducibility 4

BaSISS fields 4

Micrographs 4

Methods 6

Tissue samples 6

Histopathological assessment 6

Immunohistochemistry 7

Bulk Tissue Sequencing 9

Inferring Subclone Composition and Evolutionary Histories From Bulk Genomic Data 9

Cancer Cell Fractions 10

Multidimensional Mutation Clustering 11

Principles of Phylogenetic Tree Construction 12

BaSISS and ISS protocols 13

Tissue specimens 13

Padlock probe design 13

Mutation panel designs 14

Immune panel design 14

Oncology gene panel design 15

In-situ sequencing (ISS) 16

Imaging 17

Image stitching 17

Image registration 17

Serial tissue image signal alignment 18

ISS signal deconvolution 18

Downstream Analysis 19

Allele co-occurrence analysis 19

Nuclei segmentation 19

Single cell typing 20

Digital IHC based classification 21

Spatial mapping of cancer clones 21

Core model 21

Sources of noise 23

Cellular density 24

Differential probe specificity 24

Clone allelic variations 25

Homogeneous and inhomogeneous background adjustments 25

Sampling fluctuations 26

Optional inputs 26
VAFs from adjacent slices 27
IHC based cell type counts 27
Multi-sample version 28
Inference 29
Model limitations and assumptions 29
Spatial correlation 30
Copy number variation and expression 30
Probe affinities 30
Homogeneity of clonal expression 31
Phylogenetic tree 31
Confidence intervals of the inference 32
Clone neighbourhood analysis 32
GLMM for quantitative feature comparison 33
Inference and statistical testing 34
LCM WGS Validation 35
Mutation timing estimates 36

Supplementary Discussion Notes 36

References 38

Supplementary Tables

Supplementary Table 1: Padlock probe designs and BaSISS genotype matrices

Supplementary Table 2: Clinical histopathological information

Supplementary Table 3: Bulk WGS data; BaSISS panel mutations, clusters and signatures

Supplementary Table 4 BaSISS and ISS performance metrics

Supplementary Table 5 LCM WGS validation data

Statistics and Reproducibility

BaSISS fields

BaSISS analysis of tissue samples P1-D1, P1-ER1 and P1-ER2 shown in Fig. 2d,e; 3a, 4a; Extended Data 5b,7e were independently experimentally replicated on a consecutive slide with similar results which are presented in Extended Data 4b,d,e. P2-TN2 shown in Extended Data Fig. 6b was performed twice wihtthe same result. BaSISS analysis of tissue samples P1-D2, P1-D3, P2-TN1, P2-LN1 shown in Fig. 3e, 5a and Extended Data Fig. 6b;7a,d;9a were performed only once.

Micrographs

Fig. 3a - Single representative microregions from a total of n=3 hyperplastic, n=23 DCIS and n=19 invasive cancer regions.

Fig. 3c - representative microregion from n=1 DCIS with P1-orange (P1-ER1), n=1 DCIS with P1-red (P1-ER1), n=2 Invasive with P1-red (P1-ER1) microregions.

Fig.3d - representative microregion from n=1 DCIS with P1-green (P1-ER2), n= 2 DCIS with P1-purple (P1-ER2) and n=3 invasive with P1-purple (P1-ER2) microregions.

Fig. 4e - representative microregions of n=41 P1-green and n=42 P1-orange areas in sample P1-D1

Fig. 5c - representative microregions: n=1 of 14 with M-GP1 phenotype and of n=1 out of 8 with M-GP2 phenotype.

Extended Data Fig. 4a - n=4 example microregions of 20 LCM cuts from samples P1-D1, P1-D2, P1-ER1 and P1-ER2.

Extended Data Fig. 5d - n=1 representative microregions of n=3 hyperplastic, n=2 DCIS with P1-green, n=15 DCIS with P1-purple and n=19 invasive areas with P1-purple in P1-ER2.

Extended Data Fig. 7c - n=1 representative microregions of at least n=5 examples where mixed clones are detected within an anatomical space and a clear border is observed (in P1-D1 and P1-D2 samples).

Extended Data Fig. 7d - n=2 out of 5 monoclonal lobules and n=1 out of 5 polyclonal lobules in P1-D2.

Extended Data Fig.9a-d - representative micrographs, n =3 out of 14 M-GP1 and n=3 out ot 8 M-GP2 phenotype.

Methods

Tissue samples

Breast tissue samples were obtained from mastectomies performed for the diagnosis of multifocal primary breast cancer. Patients provided written informed consent for tissue and clinical data use in research studies. Samples and data were obtained and managed in line with the declaration of Helsinki under “project SHARE” #93-085, approved by the Dana-Farber Harvard Cancer Center Institutional Review Board. Sample and data handling at the Wellcome Sanger Institute, Cambridgeshire, UK was performed under the wider framework and approval for the Breast Cancer Genome Analyses for the International Cancer Genome Consortium Working Group under REC reference: 09/H0306/36 (Cambridgeshire 3 Research Ethics Committee). The study was later transferred to a protocol REC: 20/PR/0905 approved by London-Harrow Research Ethics Committee.

Tissue blocks were first sliced to obtain sufficient nucleic acids for bulk whole genome sequencing (WGS) and RNAseq analysis. Subsequent serial 10um sections were obtained from the same tissue block and orientation for base specific in-situ sequencing (BaSISS), in-situ sequencing (ISS), histological assessment and immunohistochemistry.

Histopathological assessment

For each tissue block a frozen section was prepared for pathological review using standard haematoxylin and eosin (H&E) staining. H&E review and annotation was performed by experienced breast histopathologists. Annotation of more than 300 microregions was performed without any prior knowledge of the genetic clonal structure (Supplementary Table 2). For DCIS annotation, regions were pre-selected for annotation to enrich for those that are predicted to be contained within a myoepithelial bordered space (ducts/acini or lobules) based on the H&E and/ or ISS histological images ("is single")

(Extended Data Fig. 4a; Supplementary Table 2). Additional regions were selected to ensure comprehensive review of the features of the tumour. Tissue sections were fresh frozen so some features such as mitoses and chromatin structure were difficult to assess and not presented in the main analyses. Consequently, nuclear grade in DCIS is largely based on nuclear size. Nuclear vacuoles were defined as present or absent based on a 20% cut-off. Differentiating between a TDLU and a duct is not always obvious on a single section – larger DCIS close to terminal structures were marked as a TDLU. Structures that are not branched and are more peripheral are defined as ducts. Lobule contours as indicated in Fig. 4a were predicted based upon spatial proximity, shared surrounding structures and/or interconnections between glandular structures observed across the available z-stack of serial tissue sections. In P1-ER1 and P1-ER2 microregions were either discrete regions such as DCIS or hyperplastic ducts while similarly sized regions of invasive tissue were demarcated manually to permit sampling across the entire tissue surface area (Extended Data Fig. 5b).

In both P2-LN1 and P2-TN1 the histopathologist independently reported the existence of more than one growth pattern. In P2-LN1 the pathologist defined the selected areas according to these growth patterns, aiming to generate similar sized regions (Fig. 5a). In P2-TN1 where there was a less clear demarcation of the different growth structures the proportion of cells belonging to each growth pattern were reported in the predefined regions (Extended Data Fig. 6b, Supplementary Table 2).

Immunohistochemistry

For immunohistochemical staining after ISS, cover glasses were detached by overnight incubation in TBS-Tween20 (0.05%). Slides were fixed for 10 min with 3.7% formaldehyde (Sigma Aldrich, Munich, Germany). Endogenous peroxidases were blocked with DAKO REAL peroxidase-blocking solution (Agilent, Glostrup, Denmark) for 10 min at room temperature, followed by incubation with DAKO serum-free protein blocking solution (Agilent) for 30 min.

Sections were incubated with mouse monoclonal PanCK antibody (clone AE1/AE3, Agilent) diluted 1:100 in DAKO REAL antibody diluent (Agilent) overnight at 4°C in a humidity chamber. Thereafter, ready-to-use secondary ImmPRESS HRP Anti-mouse IgG (VectorLaboratories, Orton Southgate Peterborough, UK) reagent was applied for 30min and chromogenic visualisation was performed with DAB Peroxidase substrate kit (Vector laboratories) according to the manufacturer's instructions. Slides were counterstained with Mayer's HTX Haematoxylin (Histolab, Gothenburg, Sweden) for 30 seconds, dehydrated and permanently mounted (Vecta Mount, Vector laboratories). A similar procedure was performed for CD45 (2B11+PD7126, mouse monoclonal DAKO Agilent; dilution 1:100, detection with secondary ImmPRESS HRP Anti-mouse IgG) and HER2 (D8F12, rabbit monoclonal; CellSignalling dilution 1:50, detection with secondary ImmPRESS HRP Anti-rabbit IgG (Vector Laboratories)).

Standard immunohistochemistry staining on non-ISS sequenced sections was performed for the following proteins: SM-MHC (BioCare Medical LLC, cat# CM 420B, mouse monoclonal, clone: SMMS-1, dilution 1:100), P63 (BioCare Medical LLC, cat# CM 163C, mouse monoclonal, clone: BC4A4, dilution 1:150), PR (DakoCytomation, cat# M3569, mouse monoclonal, clone:PgR636, dilution 1:75), Ki67 (DakoCytomation, cat#M7240, mouse monoclonal, clone: MIB-1, dilution 1:400) and PTEN (Abcam Anti-PTEN antibody [EPR22636-122] (ab267787) 1:500 pc). Secondary antibody detection was performed using polymer-M (Labelled polymer-HRP anti-mouse: DakoCytomation, Code: K4007) for PR, Ki67, P63 and PTEN (EPR22636-122) and poly-AP-M (Poly-AP anti-mouse IgG : Leica, Cat# PV6110) for SMMHC and Labelled polymer-HRP anti-mouse: DakoCytomation, Code: K4007) for PR, Ki67, P63 and PTEN (EPR22636-122) and poly-AP-M (Poly-AP anti-mouse IgG : Leica, Cat# PV6110) for SMMHC.

In selected regions (with LCM WGS validated subclone compositions) the total number and number of positive stained nuclei (Ki-67, PTEN and PR antibodies) were counted using Qupath digital software (Version 0.3.0)[1] (Supplementary Table 2).

Bulk Tissue Sequencing

To generate DNA and RNA sequence data 10 x 10-20um serial slices of tissue blocks were pooled and homogenised followed by nucleic acid extraction. Short insert whole genome and targeted capture paired-end libraries and 350bp poly-A selected RNA libraries were created, flow cells prepared and sequencing clusters generated according to Illumina protocols[2]. 100 base whole genome sequence data, 150 base targeted capture genomic data and 75 base RNA sequence data was generated using Illumina HiSeq 2000® genome analysers. Genomic and RNA sequence data were mapped to the reference human genome (GRCh37 Ensembl 58) using Burrows Wheeler Aligner[3] and TopHat (v1.3.3)(http://ccb.jhu.edu/software/tophat/index.shtml), respectively. Original data associated with the prior publication datasets from which these samples derive are deposited in the European Genome Phenome Archive (EGA) with the following accessions: EGAD00001002696 and EGAD00001000898. Bulk sequence data metrics are reported in Supplementary Table 3.

Somatic Mutation Identification

Genome-wide somatic substitutions were called using CaVEMan (Cancer Variants Through Expectation Maximisation: http://cancerit.github.io/CaVEMan/). Rearrangements were identified from discordantly mapping reads using BRASSII (BReakpoint AnalySiS) (https://github.com/cancerit/BRASS).

Inferring Subclone Composition and Evolutionary Histories From Bulk Genomic Data

Subclone composition was determined from multi-region WGS somatic substitution and copy number data as reported in the original publications[4,5]. The approach detects a finite number of subclones each with defined parameters including the number of mutations within that subclone and the fraction of

cells from each sample that contain those subclone specific mutations – the results for each sample are summarised in Supplementary Table 3.

Cancer Cell Fractions

For bulk genomic sequence data the variant allele fraction (VAF) of each mutation, i, is calculated from the number of reads reporting the mutant r_{mut} and the number of reference, or wild-type reads r_{wt} is calculated as

[1] $$VAF_i = \frac{r_{mut,i}}{r_{mut,i} + r_{wt,i}}$$

However, the VAF cannot be used directly to infer subclonal architecture because in addition to reflecting the proportion of cells that carry that mutation it is also influenced by the tumour purity and the local copy number – with the latter encompassing both the total number of alleles and the number of alleles that carry the mutation[6]. Consequently, mutations from the same subclone may have different VAFs reflecting their different copy number states, also termed their "multiplicity". To allow a comparison of mutations across related samples with different copy number profiles and tumour purity levels we derive the mutation copy number. For mutation i, the mutation copy number n_i is obtained as

[2] $$n_i = VAF_i \frac{1}{\rho} \left[\rho n_{locus,t} + n_{locus,n} (1 - \rho)\right]$$

Where ρ is tumour purity, and $n_{locus,t}$ and $n_{locus,n}$ are the locus specific total copy numbers in the tumour and normal sample respectively. Subclonal copy-number changes and tumour purity are defined using the Battenberg algorithm as previously described (https://github.com/cancerit/cgpBattenberg). Confidence intervals for mutation copy numbers are generated using bootstrap resampling of mutant and wildtype reads from each mutant locus (10,000 times) and applying the above formula to resampled reads.

For mutations that are present on multiple alleles arising through duplication (multiplicity ≠ 1) or in regions of subclonal copy number change, the mutation copy number may not accurately reflect the fraction of cells in which the mutation resides. To group mutations by the number of cells containing

those mutations, the number of chromosomes bearing a mutation, n_{chr}, must be determined. For each mutation within a region of amplified major copy number C, the observed number of mutant and wild-type reads are compared to the expected VAF_i that would result from the mutation being present in 1,2…C chromosome copies also allowing for non-integer values in regions with subclonal copy number values, assuming a binomial distribution. The value of n_{chr} is determined to be that with the maximum likelihood. The fraction of cells bearing mutation *i*, termed the cancer cell fraction or *CCF* can then be calculated as

[3] $$CCF_i = \frac{n_i}{n_{chr}}$$

Applying this approach to cancers that are believed to derive from a common ancestor, one would therefore expect to identify some clonal mutations that are present in all cancer cells and at a certain multiplicity result in a CCF around 1.

Multidimensional Mutation Clustering

A Bayesian Dirichlet Process was used to model clusters of clonal and subclonal point mutations to permit the inference of the number of subclones and the fraction of cells (CCF) within each subclone. The original model was developed for single samples[7]. Within this model the number of reads y, bearing the ith mutation is drawn from a binomial distribution as follows

[4] $$y_i \sim Binom(\pi_i,\, N_i)\ \pi_i \sim DP(\alpha P_0)$$

Where N_i is the number of mutant reads and ς_i is the expected fraction of reads that would report a mutation if it was present in 100% of cells (at that specific locus given the copy number state and tumour purity). The fraction of tumour cells carrying mutation i, π_i, is modelled as arising from a Dirichlet process (DP) with a given probability distribution P_0 and a dispersion parameter α. The approach allows co-estimation of the number of cellular populations and their properties using the stick-breaking representation of the Dirichlet process as described in Dentro et al.[6,8]

To accommodate multi-region samples, the model is extended into multiple dimensions as previously described[5,8]. Essentially, the number of mutant reads obtained from different tumour region samples are modelled as independent binomial distributions with independent π drawn with a Dirichlet process. Gibbs sampling is used to estimate posterior distributions of the features of interest. The Markov chain is run for 10,000 iterations (excluding the first 2,000 iterations). The median densities for CCFs is estimated using a Gaussian kernel implemented in R (stats and kernSmooth libraries). The clustering procedure is implemented in the DPClust software package (available here: https://github.com/Wedge-Oxford/dpclust). Version 2.2.8 was used to obtain the analysis presented.

In P2 (samples P2-TN1, P2-TN2, P2-LN1) the final DPClust solution identified a single subclone within the lymph node sample (P2-LN1) however there was evidence supporting the possible existence of a subclone albeit with lower confidence, understood in the context of low tumour purity (~11%). This is most convincing from copy number analysis that identified multiple sub-chromosomal copy number changes (chromosome 1,9, 11,12,20) in addition to a 50% subclone at the telomeric portion of 17q in association with the *HER2* amplifying breakage fusion bridge event (Figure 5B). This was further supported by a build-up of posterior density in P2-LN1, indicating that across some iterations DPClust has explored the presence of a subclone in this sample was explored, although ultimately it decided the subclone wasn't required to explain the observed point mutation data.

Principles of Phylogenetic Tree Construction

To infer the evolutionary relationship between subclones, phylogenetic trees were constructed using the "pigeonhole principle" which states that if there are *n* objects that are placed in *m* containers if $n>m$ then at least one container must contain more than one object. Extending this to subclonal reconstruction we can appreciate that the sum of subclone CCFs cannot exceed the CCF of their ancestor. For example, if there are 3 subclones with CCFs of 1, 0.9 and 0.5 then there must be a linear evolution – as 1 + 0.9 > 1, CCF subclone 0.9 must be a descendant of the CCF subclone 1; furthermore, as 0.9 + 0.5 > 1, CCF subclone 0.5 must be a descendant of the CCF subclone 0.9. In contrast one may see the possibility of branching evolution where the sum of subclone populations is less than 1. For example, 3 subclones

with CCFs of 1, 0.5 and 0.4 could arise either through linear evolution: 1 > 0.5 > 0.4 or along separate ancestral lines because 0.5 + 0.4 < 1. When applying these principles across multi-region samples the compatible underlying tree structures is usually greatly restricted because of the requirement that the same tree structure must be compatible across all samples. Applying these principles one can identify the phylogenetic tree structure(s) most compatible with the underlying data.

BaSISS and ISS protocols

Tissue specimens

Serial 10um tissue sections were cut from fresh frozen tumour tissue blocks mounted on superfrost slides and sent from DFCI to ScilifeLab Stockholm, Sweden where ISS and IHC was performed under Karolinska Institutes rules for the handling of blood and other human sample material, reference number 1-31/2019 (with a local HUMRA risk assessment form). Tissue blocks included the same ones used for the WGS and RNAseq experiments and additional tumour blocks were identified where applicable.

Padlock probe design

The padlock probe consists of a single stranded DNA oligo with two target recognition arms in the 3' and 5' end of the oligo, enabling circularization of the padlock probe upon target hybridization. Each target recognition arm typically has a length of 15-25 nucleotides. The two arms are interspaced with a linker sequence comprising a 20-nucleotide anchor primer sequence and a target-specific 4 nucleotide barcode followed by a 5 nucleotide stabilising sequence for sequencing-by-ligation. The used barcodes were selected in such a way that they differ in at least two positions.

In total, three padlock probe gene panels were used, marker/oncology gene-, mutation- (one specifically designed for each case) and immune- expression panels (Supplementary Table 1). Padlock probes were

ordered as ultramer DNA oligos from Integrated DNA Technologies (IDT, Leuven, Belgium) with 5'-phosphorylation modification and were reconstituted in Tris/EDTA buffer.

Mutation panel designs

Two separate case-specific mutation panels were designed as detailed in Supplementary Table 1. For wildtype- and mutation-specific padlock probes, the sequence upstream of the mutated site resembles the 3' arm of the padlock probe, with the wildtype or mutant nucleotide at the 3' end, whereas the sequence downstream of the mutated site resembles the 5' arm. The target recognition arm lengths were adjusted to have a similar melting temperature of sim ~55°C. For each mutation site, one wild-type and one mutation-specific padlock probe was designed. For P1, in addition to the anchor and barcode sequences a 20-nucleotide sequence used for a hybridization cycle was added in the padlock probe linker sequence. Specific primers were used for the in situ reverse transcription and were designed as the reverse complement sequence of the 5' target recognition arm. Primers were ordered as DNA oligos from Integrated DNA Technologies (IDT) and were reconstituted in Tris/EDTA buffer. In P1, we included 3 mutations that were not assigned to a cluster/ branch by DPclust of WGS data. In accordance with this, BaSISS signals reporting these mutations were found to be non-specific and uninformative, probably relating to them existing at loci with variable copy number states, and they were dropped from further downstream analyses.

Immune panel design

Large scale probe design was facilitated using an in-house Python software package as described previously (Ref PMID 31740815) which utilises ClustalW and BLAST+ to ensure probe specificity. Each padlock probe of the immune panel was designed to contain two 20 nucleotide long target recognition arms. Only target fragments with melting temperature between 65°C and 75°C were considered. Probes were selected aiming to obtain a distribution along the whole length of the transcript.

Overall, five padlock probes were selected per target gene with the exception of *HLA-DRB1*, where only two specific probes could be designed. For the T cell specific genes *CD8A, FOXP3, EOMES, CD4* and *IFNG* 20 probes were selected in order to increase the detection efficiency for these target genes. All padlock probe sequences of the immune panel are shown in Supplementary Table 1.

A combination of random decamer primers (IDT, Leuven Belgium) and specific primers were used for in situ reverse transcription. Specific primers were designed to hybridise to the mRNA 15-20 nucleotides downstream of the target sequence. Primers were ordered as DNA oligos from Integrated DNA Technologies (IDT) and were reconstituted in Tris/EDTA buffer.

Target genes for the immune panel were selected to cover a broad range of immune cell subtype markers with special emphasis on T cell subsets and their regulation (Supplementary Table 1).

Oncology gene panel design

The oncology gene panel has previously been published and includes genes involved in proliferation, EMT, invasiveness, stemness, angiogenesis as well as genes for breast cancer subtyping and oncotypeDX recurrence scoring[9]. The target recognition arms were designed to capture most splice variants of the gene transcripts and blasted to confirm their specificity. Each target recognition sequence had a GC content of 50-55% and a melting temperature of ~55°C. In this older design, the panel included one padlock probe per gene target and the barcodes used were only differing in one position. For the marker gene panel, random decamer primers were used for *in-situ* reverse transcription (IDT, Leuven Belgium).

In-situ sequencing (ISS)

ISS was performed as described by Ke et al.[10] and modified according to Svedlund et al.[9], and was used to spatially resolve oncology panel-, mutation panel- and immune panel- gene expression profiles on consecutive sections from the breast tumour tissue blocks.

The ISS library preparation and sequencing is described in detail at protocols.io.bb2giqbw, the steps in the protocol that were modified for breast cancer tissues are indicated below. In brief, library preparation included fixation of tissue sections with 4% PFA for 30 min (step 2) followed by permeabilized with 0.1 mg/ml pepsin (Sigma) in 0.1 M HCl 37°C for 90 s (step 4). SecureSeal™ reaction chambers were mounted on top of the tissues (Grace Biolabs, Bend, United States) and cDNA was synthesised *in situ* using specific DNA primers (125nM for mutation panel, 5nM for immune panel) and/or random decamer primers (5µM for immune and marker gene panels) (step 10) (IDT, Leuven Belgium, sequences are listed in Supplementary Table 1). Rnase H was used to generate single-stranded cDNA that the padlock probes could hybridise to. Hybridized padlock probes (10nM of each in immune panel, 0nM of each in mutation and marker gene panel, step 15) (IDT, Leuven Belgium, sequences are listed in Supplementary Table 1) were ligated using Tth ligase, a highly specific DNA ligase that can discriminate correct base-pairing at the single nucleotide level. Only completely target-complementary padlock probes become ligated, forming closed circles that could then be amplified through rolling circle amplification (RCA). Of note, for P1, the experimental conditions for the first replica of ISS differed slightly with a diverse Phi29 buffer (Thermo Fisher 10X reaction buffer: 330 mM Tris-acetate (pH 7.9 at 37°C), 100 mM Mg-acetate, 660 mM K-acetate, 1% Tween 20 and 10 mM DTT) and no Exonuclease 1 in the rolling circle amplification step.

The four-five nucleotide target-specific barcodes included in the padlock probe linker sequence were clonally amplified in the RCA products allowing identification through sequencing by ligation (according to Dermat et al.) of anchor primer and fluorophore-labelled interrogation probes [9]. Nuclei were stained with 4',6-diamidino-2-phenylindole (DAPI). The target-specific barcodes were sequenced

with four sequencing and imaging rounds. For case 1, the mutation panel was sequenced with one hybridization cycle in addition to the four sequencing by ligation cycles. After the ISS analysis, the tissue sections were stained with PanCK, CD45 or HER2 antibody.

Imaging

Images were acquired with an automated Zeiss Axioplan II epifluorescence microscope (Zeiss, Oberkochen, Germany) using a z-stack of 0.49 µm × 11 and a tile overlap of 10%. Images were scanned with a 20X objective. For the first base sequenced, the exposure times were calibrated so that the signal intensity values were similar for all sequencing channels (A–Cy5, G-Cy3, C-Texas Red and T-AF488), the calibrated exposure times were then kept constant for all remaining sequencing cycles. Orthogonal projections and stitching of tiles were done with the ZEN software (Zeiss).

Image stitching

From a total of 51 image sets, 43 were stitched with Carl-Zeiss ZEN software (version 3.1), and the other 8 failed image sets were stitched using BigStitcher (version 0.9)[11].

Image registration

The registration across imaging cycles was performed in two steps: affine registration on DAPI channel and subsequently local warping on anchor channel. For both steps we used algorithms provided in libraries OpenCV-contrib (version 4.3.0)[12] and scikit-image (version 0.17)[13]. In all imaging cycles, before the registration, both DAPI and anchor channels were maximum intensity projected across the image z-stack.

During the affine registration step, we coarsely align images of all cycles to the first one based on the DAPI channel. Firstly, we detect key points in the images of each cycle using the FAST feature detector. Secondly, for each key point, its surrounding area is described with histograms of oriented gradients using the DAISY feature descriptor. After that, using the key points and their descriptors, the FLANN-

based matcher finds correspondences between pairs of key points from reference and moving images and filters out unreliable points. Lastly, the remaining key points are processed using the RANSAC-based algorithm that aligns them and estimates affine transformation parameters with 4 degrees of freedom.

The second registration step aligns imaging cycles sequentially using the anchor channel (fluorophore Cy7). We applied the Farneback optical flow algorithm to achieve more accurate registration by warping the images locally, so that RNA spots of different channels can be better aligned despite the presence of nuclei swelling, imperfect stitching and sample distortion. In both steps, we optimised the algorithm by performing computation on the tiled images to reduce memory consumption and accelerate the transformation parameters estimation.

Serial tissue image signal alignment

Analysis of sample P2-LN required IHC signal projection performed on a consecutive slide back to BaSISS slide. To achieve this, we performed a spline-based elastic registration implemented in ImageJ package UnwarpJ[14].

ISS signal deconvolution

After registration of images from different sequencing rounds, we locate RNA spots by applying the circular Hough transform to the reference anchor channel of the first round, which is implemented in MATLAB's function 'imfindcircles'. At the detected coordinates, image values are extracted from top-hat filtered coding channels across all sequencing rounds. We then perform decoding of the extracted image values via a Gaussian Mixture Model, where each mixture corresponds to one of the possible barcodes encoded via an experimental design. Finally, once the mixture model is fitted to the extracted image values, each detected spot is assigned to the most likely barcode[15]. In addition to on-target barcodes, there is an infeasible class which represents RNA spots to which barcodes could not be

assigned. In Supplementary Table 4 we present QC metric of our datasets as a proportions of RNA spots with assigned barcodes ($p > 0.6$) to the total number of spots.

Downstream Analysis

Allele co-occurrence analysis

The relative co-occurrence of BaSISS alleles was calculated using the M-function[16] at a 20μm radius. Randomised point types (points have a fixed location but labels are resampled) were used as a null-hypothesis and 200 samples were used for the confidence number estimation. The exact score is computed as the deviation of the observed value from the simulation mean, normalised by the spread of the confidence intervals at the α = 0.005:

$$Score = \frac{M(obs) - M(sim)_{0.5}}{0.5*(M(sim)_{0.5} - M(sim)_{0.005} + M(sim)_{0.995} - M(sim)_{0.5})}.$$

Inference and confidence interval estimation was performed with dbmss R package[17]. For LCM allele co-occurrence a simple Pearson correlation of variant allele frequencies between regions was used.

Nuclei segmentation

A two-stage pipeline from Caicedo, Goodman et al.[18] was used for nuclei segmentation of a DAPI image. First, an ensemble of 32 pre-trained neural networks was used to classify image pixels into three classes: background, nuclei and nuclear boundaries. The ensemble included neural networks with UNet- and FPN-like architectures with the following encoders: DPN-92, Resnet-152, InceptionResnetV2, Resnet-101. Then, the individual nuclei masks were obtained using the watershed algorithm. For each nucleus candidate, a set of morphological features (e.g. circularity, convexity, area, neighbour's median area) was computed. After that, a gradient-boosting model (LGBM) based on these features was used

to filter real nuclei from false-positive predictions. Code is available at github.com/yozhikoff/segmentation.

Single cell typing

The decoded ISS signals do not directly reveal which cells they derive from require further processing to recover these associations. To do so, ISS data was overlaid with the nuclei segmentation masks and signals were assigned to the nearest nuclei. A conservative distance cut-off of 5µm was used to decrease the chance of missannoation which led to the ~30% of signal loss.

Since ISS data is sparse and restricted to a limited number of targeted genes, a previously published scRNA breast cancer atlas[19] was used for the label transfer. The differences in gene detection rates between scRNA reference and ISS data combined with the low signal per nucleus counts, limited the scope of applicable computational approaches. Therefore, the label transfer was based on the most informative marker genes and was performed in a hierarchical manner as follows.

Single cell annotation data often has hierarchical structure reflecting the hierarchical nature of the cell differentiation. We could leverage this structure as a way of regularisation for the cell type prediction model where the fine cell subtyping (e.g. CD4 T-cell) is dependent on the top annotation level (e.g. T-cell). A hierarchical multinomial logistic regression model trained on the reference scRNA dataset restricted to the genes available in the ISS panel would encode the gene importance for the cell type characterisation on a respective level of hierarchy. We used this approach to assist marker gene selection and arrived with the panel of genes for broad (Immune, Epithelial and Stromal) and specific (B-cells, Myeloid, T-cells, Fibroblasts + PVL and Endothelial) cell type levels (Extended Data Fig. 2b,c). The code is available at github.com/dissatisfaction-ai/scHierarchy.

The final cell type assignment was performed in 2 steps. At first iteration, nuclei were classified into 3 broad categories (‘epithelial’, ‘immune’, ‘stromal’) if they had any corresponding marker genes

assigned. At the second iteration, nuclei with 'immune' and 'stromal' assignments were further subdivided into specific groups based on the presence of the corresponding markers. The identity of nuclei that did not have any marker genes in proximity or had a contradictory assignment was considered 'unknown'.

Digital IHC based classification

To quantitatively describe DAB-stained areas, Haematoxylin-Eosin-DAB colour deconvolution was used[20]. The implementation from the scikit-learn Python package was used.

Spatial mapping of cancer clones

Raw BaSISS data informs us of allelic variant's spatial distributions. However, this data cannot be directly interpreted in terms of cancer clones. To make sense of BaSISS data we designed a Bayesian model that aims to decompose local BaSISS signal counts into clone densities and corresponding genotypes.

Core model

The BaSISS protocol records a series of fluorescent spots, which are decoded into a class of different barcodes A corresponding to each targeted allele. This information produces a table of tuples (a_i, x_i, y_i), where a_i is the allele of spot i and x_i and y_i are its respective two-dimensional coordinates.

The counts of all probes can be represented by a three-dimensional array $D \in N^{|a|\times|x|\times|y|}$, where a refers to the allele and x and y are coarse grained coordinates on a regular grid of dimensions $|x| \times |y|$. The grid size was chosen to be 108.8μm considering a trade-off between data sparsity, precision and computational cost.

The essential idea is that the expected number of BaSISS signals is decomposed into maps of $|s|$ distinct clones s $M \in R^{|s|\times|x|\times|y|}$ each with a distinct genotype $G \in N^{|a|\times|s|}$,

$$E[D] \approx G \times M = \sum_{s \in subclones} G_{\cdot,s} M_{s,\cdot,\cdot}$$

The genotype matrix G contains the number of each allelic copy in each clone s. G is a matrix representation of the underlying phylogenetic tree and provides the instructions of the allelic configuration in each branch of the tree.

The maps M provide the relative prevalence of each clone in a given area of the grid. As M should replace a spatially continuous map, it is modelled by softmax transformed two dimensional latent Gaussian processes.

Latent Gaussian Process modelling of spatial clone maps

Depending on the choice of targeted alleles, signal counts in matrix D may appear rather sparse. For a robust clone prevalence map inference, we use Gaussian processes as it allows us to naturally utilise spatial distance information. Each clone s is modelled as independent latent two-dimensional Gaussian process with $\mu = 0$ and covariance matrix K with RBF kernel over grid coordinates. Bandwidth l was empirically chosen for sample P1 and adjusted for sample P2 according to the median signal/nuclei ratio.

$$K_x(x, x') = exp\left[-\frac{(x - x')^2}{2l^2}\right]$$

$$m_s(x, y) \sim GP(0, K_x \otimes K_y)$$

Each Gaussian process was mapped to simplex space using a softmax (multidimensional logistic) transformation with temperature parameter $\tau = 2$ which represents our weak prior belief that clones could cover the whole range of $[0,1]$ prevalence.

$$M = \left[\frac{e^{\tau m_0}}{1 + \sum_{i=0}^{|s|-1} e^{\tau m_i}}, \dots, \frac{e^{\tau m_{|s|-1}}}{1 + \sum_{i=0}^{|s|-1} e^{\tau m_i}}\right]$$

Sources of noise

To fit the above model to data a number of sources of noise need to be considered.

1. Cellular density ν
2. Differential probe specificity ι
3. Allelic confusion τ
4. Clone expression variations γ
5. Homogeneous and inhomogeneous background adjustments β
6. Overdispersed sampling fluctuations α

Accounting for these sources of noise, the equation for the expected number of BaSISS signals $\mu = E[D]$ becomes:

[1] $$\mu_{a,x,y} = \underbrace{\nu_{x,y}}_{\text{cell density}} \cdot \overbrace{\iota_a}^{\text{detection rate}} \cdot \underbrace{\sum_{a'} \tau_{a,a'}}_{\text{probe confusion}} \overbrace{\sum_s \gamma_{s,a}}^{\text{clone-specific expression}} \underbrace{G_{a,s} M_{s,x,y}}_{\text{clone contribution}} + \overbrace{\beta_a}^{\text{background}}$$

The exact nature of and statistical models for the different noise terms will be discussed in the next subsections.

Cellular density

The prevalence of clones is most insightful when expressed as the density of cells per area. A DAPI stained image of nuclei is obtained as part of the BaSISS experimental workflow and utilised to identify nuclei. We use a neural network ensemble model to segment these images and store nuclei counts in a two-dimensional array $N \in N^{|x| \times |y|}$. However, these counts might not always correctly quantify the true cell densities due to possible segmentation errors and the fact that only a fraction of cells might be captured on the slide. Therefore, we treat the nuclei counts $n_{x,y}$ as a Poisson distributed values with mean $\nu_{x,y}$ representing cell densities which in turn has a weak Gamma prior.

$$N_{x,y} \sim Poisson(\nu_{x,y}),$$

$$\nu_{x,y} \sim Gamma(\mu = 50, \sigma = 100).$$

Differential probe specificity

BaSISS' ability to detect allelic variants relies on a multistage process of reverse transcription, padlock probe annealing, amplification and fluorophore detection. The resulting intensity will be the product of these steps and differ between each probe/allele. As only the product of all steps can be identified, we model the variant detection rate $\iota \in R_{+}^{|a|}$ for each allele a

$$\iota_a \sim Gamma(\mu = 0.5, \sigma = 1).$$

Allelic confusion

The difference between wild type and mutated allelic variants is often just a single nucleotide. This increases chances for the padlock probe to anneal to the wrong allelic variant resulting in allelic confusion. To model this confusion, we design a sparse transition matrix $\tau \in R_{(0,1)}^{|a|,|a|}$ populated with

transition probabilities $\{\tau_{a,a'}\}$ for each allele. We put a strong regularising Beta prior on $t_{a,a}$ to shift probability density close to *1* and prevent it going below *0.75* to avoid non-identifiability.

$$\tau = \begin{pmatrix} & \dots & wt & mut & \dots \\ \vdots & & & & \\ wt & & \tau_{11} & 1-\tau_{11} & \\ mut & & 1-\tau_{22} & \tau_{22} & \\ \vdots & & & & \end{pmatrix}$$

$$\tau_{a,a'} \sim 1 - \mathrm{Beta}(\alpha = 1,\ \beta = 25)/4$$

Clone allelic variations

Although the mean clonal expression level is registered in detection rate matrix ι, it is reasonable to assume that different clones may have some level of diversity in expression of some genes. We model this deviation as a matrix $\gamma \in R_{+}^{|s|\times|a|}$ with a log-normally distributed prior such as $E[\gamma] = 1$.

$$\gamma_{s,a} \sim LogN(\mu = 0, \sigma = 0.05)$$

Homogeneous and inhomogeneous background adjustments

Raw BaSISS data comes from biological images which have additional sources of variability. The first one is the homogeneous additive effect $\beta \in R_{+}^{|a|}$ which increases the expected value of a particular probe detection globally on the slide.

$$\beta_a \sim Gamma(\mu = 0.5, \sigma = 1)$$

The second one is a spatially inhomogeneous background which adjusts for local base signal detection variability. In practice it's hard to reliably model this type of background without a harsh regularisation due to its flexibility. We encode this inhomogeneous background as additional pseudo-clones p which have corresponding relative prevalence M and pseudo-genotype $\Gamma \in R_{+}^{|p|\times|a|}$. Prevalence is modelled together with the real clones as a two-dimensional latent Gaussian process. The pseudo-genotype is sampled from a Beta prior which support is stretched to match median copy number k among the loci. We use $|\psi| = 1$, but it can be increased for a more flexible background effect.

$$\Gamma_{p,a} \sim Beta(\alpha = 0.01, \beta = 1) \times k.$$

Sampling fluctuations

We model BaSISS counts as Negative Binomial distributed given the expected detection level $\mu_{x,y,a}$ defined in Eq. (1) and overdispersion parameter α_a which accounts for unexplained homogeneous variance:

$$D_{x,y,a} \sim NB(\mu_{x,y,a}, \alpha_a).$$

The over-dispersion α_a is sampled from Gamma distribution where distribution with mean 100 and a standard deviation of 10

$$\alpha_a \sim Gamma(\mu = 100, \sigma = 10).$$

Optional inputs

Depending on which additional data accompanies investigated samples we may want to include them into the proposed Bayesian framework. Currently, two options are implemented:

1. VAFs from adjacent slices
2. IHC based cell type counts

VAFs from adjacent slices

WGS data contains information of the mutated allele frequencies on the whole slide. We can use this data to increase the accuracy of our inference and stabilise the overall prevalence of clones. To pass it into the model we calculate the expected aggregate frequencies of each mutated variant m on the slide, $VAF^{BaSISS} \in R_{[0,1]}^{|m|}$, by summation of $M \times G$ product over spatial dimensions.

$$VAF^{BaSISS} = \sum_{x,y} M \times \frac{G^{mut}}{G^{wt} + G^{mut}}.$$

We then construct a Beta pseudo-likelihood of VAF^{BaSISS} with α and β parameters proportional to the number of mutated and wild-type reads in the WGS experiment. We use the parameter $u = 10$ to inflate uncertainty in WGS data as it comes from proximal but not exactly the same slide, we are observing in BaSISS experiment

$$VAF_m^{BaSISS} \sim Beta(\alpha = WGS_m^{mut}/u + 1, \alpha = WGS_m^{wt}/u + 1).$$

To adjust for a disproportionately higher impact of spatial information on the total likelihood we multiply VAF log-likelihood by the number of tiles on the slide.

IHC based cell type counts

IHC data reveals part of cell type heterogeneity. In our study we mainly worked with CD45 IHC staining which indicates the location of lymphocytes. As we know that none of the lymphocytes should contribute to cancer clone weights, we could further regularise clone weights. This is done by

construction of cell fraction matrix $CellFrac \in R_{[0,1]}^{|x|\times|y|}$ obtained by summation of clone map matrix M over clones of respected type s^{-} or s^{+}

$$CellFrac = \frac{\sum_{s\in s^{-}} M}{\sum_{s\in s^{+}} M}.$$

We use a Beta pseudo-likelihood of $CellFrac$ distribution with α and β parameters equal to cell counts of clear and IHC stained nuclei on the slide

$$CellFrac_{x,y} \sim Beta\big(\alpha = IHC_{x,y}^{-} + 1, \alpha = \ IHC_{x,y}^{+}, +1\big).$$

Multi-sample version

In many cases some of the clones belonging to the same phylogenetic tree might not exist or exist only in a small proportion on most of the slides. Having a multi-sample generalisation of the proposed model helps us to correctly infer parameters associated to such clones. Essentially, the only parameters which are shared between all slides k are:

- genotype matrix G,
- probe confusion transition matrix τ
- mean probe detection rate ι and
- overdispersed sampling fluctuations α.

All the other parameters become slide specific, i.e., cellular density ν, differential probe specificity ι, clone allelic variations γ and homogeneous and inhomogeneous background adjustments β.

To allow some level of slide specific probe efficiency variation we multiply mean probe detection rate by a slide specific probe deviation matrix $\eta \in R_{+}^{|k|\times|a|}$ with a log-normally distributed prior such as $E[\eta] = 1$

$$\eta_{k,a} \sim LogN(\mu = 0, \sigma = 0.05).$$

Inference

Variational Bayesian Inference is used to approximate the posterior. We use the mean-field version of Automatic Differentiation Variational Inference (ADVI) implementation from pymc3 package [21]. Briefly, within the ADVI framework, the posterior distribution over unknown parameters is approximated by an appropriately transformed multivariate normal distribution with a diagonal covariance matrix. Inference is achieved by minimising the KL divergence between the variational approximation and the true posterior distribution which is equivalent to the maximisation of the evidence lower bound (ELBO).

Training is stopped when the ELBO stops increasing passing at least 15,000 iterations of training using the ADAM optimiser with a learning rate of 0.01. The marginalised variational posterior of the parameters was used in the downstream analysis. Our experiments showed that several initialisations may be needed to arrive at an optimal and solution. This is mostly due to the stochastic choice of the initial values but may also be influenced by numerical artefacts arising during influence with pymc3.

Model limitations and assumptions

Quantitative estimation of the spatial cellular composition of genetically distinct clones based on allelic information from in-situ RNA sequencing is a challenging problem. The aforementioned model offers a solution that deals with the multiple sources of biological and technical noise to produce clonal maps which have been orthogonally validated (see validation section). However, it comes at the cost of introduced assumptions and limitations that make the solution possible.

Spatial correlation

A Gaussian process prior is used to deal with the sparsity of the ISS data. The assumption is that the clonal composition changes smoothly across space. The biological reasoning behind it is based on the fact that breast cancer tissue exhibits some structural integrity, so when cells divide they remain close to each other. However, it's important to note that the model doesn't take into account the existence of detached compartments such as ducts. As this assumption breaks on the interface between compartmentalised regions, the inferred clonal composition on the borders may appear slightly more intermixed than it is in reality.

Copy number variation and expression

The BaSISS protocol detects RNA, however cancer clones are determined by their genotypes. To be able to conduct inference in relation to genetic clones, a relation between genotype and transcription needs to be stated. We assume a positive linear relationship between copy number of an allelic variant and its expression (and detection), with a possibility of a clone specific deviation. Previous studies suggest that the correlation between CNVs and expression seems to hold for many genes[22,23].

Probe affinities

A further technical reason that weakens the link between genotype and raw BaSISS abundances stems from the fact that ISS uses molecular probes which bind to cDNA with variable affinity. This can lead to some ambiguity as probe affinities need to be estimated from the observed data, which requires additional information. This is especially the case if there are few clone defining variants available (otherwise poorly and strongly binding probes may average out). To assist estimation of the allele and clone specific detection rate, it is instrumental to use prior information on the clonal composition, at least at the level of the whole tissue section. Given the first step in the BaSISS workflow is to perform

subclone discovery by WGS, prior knowledge of clone composition exists in most cases, even considering the possibility of sampling bias.

Homogeneity of clonal expression

Another assumption is that alleles belonging to a particular clone are expressed homogeneously across the slide. Violations of this expectation could be partly compensated by:

1. Multiple alleles defining clonal branches. If only a few alleles are highly variable, the rest could compensate for that. Overdispersion parameters would automatically decrease the influence of alleles with higher heterogeneity than expected.
2. Inhomogeneous background. Part of the heterogeneity could be captured as the background 'residual' clone.

Phylogenetic tree

The clonal genotype matrix / phylogeny is a discrete model parameter, such that the model doesn't have a way to adjust it directly. While it is theoretically possible to select an optimal tree solution based on BaSISS data, we observed that in practice different tree solutions provide very similar ELBO values, likely due to the aforementioned unknown parameters which lead to a practical unidentifiability. Therefore, attention should be paid during WGS mutation clustering and copy number estimation, as errors in these quantities may lead to unrealistic clonal abundances and growth patterns. However, assessment of the model residuals could indicate problematic alleles and indicate if the genotype matrix is feasible.

Confidence intervals of the inference

Mean-field variational approximation allows relatively fast inference but has several limitations on its own. Variational approximation of the posterior is estimated using the reverse-KL divergence $KL(Q||P)$. If the true posterior P is multimodal, the variational approximation Q is known to fit only one of the modes of P, yielding only one of the potential solutions (the likely combination of the parameter values) and missing other potential solutions. In addition, mean-field approximation has a tendency to underestimate the parameter variance[24].

Clone neighbourhood analysis

Knowing the spatial distribution of clonal densities, one may characterise the clones phenotypically based on additional spatially matched data. These include:

- histopathological phenotype,
- ISS expression signals assigned to nuclei,
- nuclei cell types and
- IHC staining.

These modalities of spatially resolved information are located on several consecutive slides of tissue which could be distorted from one layer to another. To integrate information among all the modalities, we annotated structurally similar areas of breast tissue on each slide which should be in physical proximity on the z-stack (see Histopathological assessment).

For differential phenotype analysis, highly clonal areas were selected based on the applicable threshold, $CCF_{clone} > 0.7$ for P1, and $CCF_{clone} > 0.15$ and 0.05 for P2-TN1 and P2-LN1 respectively (P2). The lower threshold for the P2-TN1 and P2-LN1 is due to a presence of infiltrations with the cells of a non-epithelial origin.

GLMM for quantitative feature comparison

Cell type specific expression

To measure cell type specific differential expression associated with clonal regions, we record ISS signals which belong to the nuclei classified as the cell type of interest. In this case, the distribution of observed ISS expression signals Y_{rg} of genes g across regions r is modelled as a result of a clone specific expression rate β_{cg},

$$\beta_{cg} \sim Uniform(-10, 10).$$

Acknowledging potential region specific expression variation, we introduce a random effect variable α_{rg} which has a clone specific hierarchical prior σ_{cg}. The binary matrix A_{rc} maps dominant clones to regions,

$$\sigma_{cg} \sim HalfNormal(0.05),$$

$$\alpha_{rg} \sim Normal(0, A\sigma_{cg}).$$

After accounting for the number of nuclei of the relevant cell type x_r, we compose a likelihood:

$$Y_{rg} \sim Poisson(x_r e^{A\beta_{cg} + \alpha_{rg}})$$

Cell type composition

To assess differential cellular composition between regions associated with the clones, we count the number of nuclei classified for each of the cell types. The distribution of nuclei counts Y_{rt}attributed to the cell type t across regions r is modelled as a mixed model of clone specific frequency β_{ct} and a region specific random effect α_{rt} , in a similar way as the *cell type specific expression*:

$$\beta_{ct} \sim Uniform(-10, 10),$$

$$\sigma_{ct} \sim HalfNormal(0.05),$$

and

$$\alpha_{rt} \sim Normal(0, A\sigma_{ct}).$$

However, the sum-to-one constraint of the cell type frequency, requires a softmax (logit) link function and Multinomial likelihood for the cell types:

$$\lambda_{rt} = A\beta_{ct} + \alpha_{rt},$$

$$Y_{rt} \sim Multinomial(n = x_r, p = \frac{e^{\lambda_{rt}}}{1+\sum_{1}^{T-1} e^{\lambda_{rt}}}).$$

IHC data

For each region, the number of IHC-positive and IHC-negative nuclei was counted using QuPath[1]. Then, IHC-positive and negative nuclei were treated as 2 distinct cell types, so the *Cell type composition* model would be applicable.

Inference and statistical testing

The models were implemented in numpyro[25] and HMC was used for the inference of the parameters. Differentially expressed genes were determined through pairwise comparisons. The difference between clone specific expression rates β_{cg} was calculated for each clone pair. As HMC produces empirical posterior distributions with finite samples the distinction of small differences is challenging and probabilities/quantiles are limited to 1/n where n is the HMC sample size and many. This becomes problematic when multiple comparisons are being made, which requires multiple testing adjustments, achieved via appropriate priors during Bayesian inference, which effectively scale the posterior. To enable calculating more extreme quantiles, the square of the mean divided by the standard deviation $(\frac{\mu(\Delta)}{\sigma(\Delta)})^2$ was used to approximate the posterior by χ_1^2 distributions (a similar approximation is

commonly made in frequentist statistics). Differential composition and protein expression were determined in the same way, with the softmax transformed β_{ct} values used instead. Treating the posterior (in a slight abuse of Bayesian principles) as analogue to a frequentist test statistic, we apply multiple testing corrections for each comparison to control the FDR < 0.1. To avoid unnecessary loss of statistical power in clone-specific differential expression analysis, genes with a clone-agnostic average number of detected signals per region < 0.3 were not considered.

LCM WGS Validation

Residual frozen tissue blocks from P1 samples P1-D1, P1-D2, P1-ER1 and P1-ER2 were accessed to perform laser capture microdissection (LCM) and low input library whole genome sequencing as previously reported[26]. Ten micrometre thick sections were generated at -20 °C and mounted on poly-ethylene naphtholate (PEN)-membrane slides (Leica), fixed with 70% ethanol for 2 minutes followed by serial immersion in the following: deionised water (1 minute), Gill's haematoxylin (10 seconds), tap water (20 seconds, twice), eosin (5 seconds), tap water (20 seconds), ethanol 70% (20 seconds, twice), ethanol 100% (20 seconds, twice), xylene (20 seconds, twice).

Using a laser-capture microscope (Leica LMD7), breast tissue architecture in relation to existing mapped clones (where feasible) was first visualised, then regions of approximately 13,000-180,000um^2 were dissected (power 55, aperture 2) and collected into the individual wells of a 96-well plate. Samples were then processed as described by Ellis et al.[26]. Briefly, individual steps include cell lysis and digestion using Arcturus PicoPure Protease buffer and thermal cycling in a sealed plate (60 °C for 3 h, 75 °C for 30 min, hold at 4 °C), gDNA purification using Agencourt AMPure beads, enzymatic DNA library construction (NEBNext Ultra II FS DNA Library Prep Kit for Illumina (New England Biolabs, cat. no. E7805L)), adapter ligation and amplification. Genomic libraries with concentrations of >5ng/ul DNA (total volume = 20ul) were selected for NovaSeq 6000 paired end sequencing. A bulk whole genome library was also generated from whole blood derived DNA and sequenced in the same way. As

described above for bulk WGS analysis, point mutations were called using CaVEMan and copy number using ASCAT and Battenberg algorithms and mutation clusters identified using DPclust v2.2.8 (Supplementary Data Table 5).

Mutation timing estimates

We report that there is evidence of early divergence of the subclones detected in P1 (prior to 50% of in m time). Evolutionary timing estimates can be sought for P1 but are not attempted for P2 due to relatively low purity. Based on the assumption of a relatively constant mutation rate within a given genetic lineage, subclone divergence as a percentage of evolutionary time can be calculated from the number of mutations accumulated up to the branching point (branch lengths) divided by the longest series of branches within that lineage. Mutation rates particularly in cancers might be greatly altered by hypermutator states, however, we confirmed that in P1 virtually all mutations in this case are derived from clock-like mutational processes using our previously published approaches (Signature 1, 5 and 40) (Supplementary Table 3)[27].

Supplementary Discussion Notes

A key observation from these data is the variable relationship between genotype and phenotype. We examined three primary breast tumours with intermixed DCIS and invasive disease, and in each case, a clone was identified that existed simultaneously in the two distinct histological states. In contrast, albeit in a single examined case, we observe remarkable consistency between genetic lineage and DCIS grade spanning centimetres of tissue. While occasional examples of 'upgrading' occur, which could be accounted for by unsampled genetic evolution, we find no evidence of transition to a lower grade. The lack of plasticity within the grade phenotype space is aligned with studies that report that DCIS and invasive cancers are usually of the same grade[28,29]. Our findings could reflect stable differentiation states acquired by divergent cells, prior to DCIS onset[30].

Another key message, that echoes those of a recent spatial study in melanoma[31], is that measuring entire tissue sections rather than small fields of view is advantageous for understanding biology. Firstly, this approach reveals that patterns of cancer growth and organisation can vary across different scales. For example, in P1-D1, P1-D2 and P1-D3, at macroscopic scale we see DCIS clones co-exist in multiple lobules, across an entire breast lobe, while at the microscopic level they are mainly segregated, distending individual acini or forming abutting populations. There are various possible explanations for the observed DCIS growth patterns such as clonal co-operation or genetic drift [32,33]. A number of recent mathematical modelling studies have emphasised the importance of tissue architecture in shaping evolution and in the reported case it is certainly plausible that altered rates of fixation and absorption arising from the physical bottlenecks in the different sized ducts and ductules could account for the observed genetic patterns[34–36]. Secondly, within a lymph node the panoramic view allows one to appreciate that subclones repeatedly foster the same ecosystems – one that is immune depleted within the sinuses while the other clustered around B-cell aggregates consistent with a clone specific interactions with the adaptive immune response[37]. This way of examining tissues naturally leads to a multitude of questions that each warrant follow up in its own extended cohort. The tools we have developed and approaches demonstrated in this study will provide others with a framework for achieving this.

Particular advantages of the technology are that it is capable of interrogating very large tissue sections and it is comparably cheap, unlike solely relying on sequencing based methods[38]. However, of course the de novo mutation detection step must also be considered in terms of the complexity and cost of the entire workflow. The granularity of clones mapped by BaSISS depends on this critical phase and can be adapted according to the precise research question. Here we used multi-region WGS (~40 fold coverage per sample) to identify and map relatively broad subclone populations but the spatial genomics approach could be applied to single genomes, high depth targeted capture approaches or more detailed phylogenies obtained through LCM or even single cell approaches. In theory, the approach also holds the potential to create three dimensional genomic tomographs by aligning

consecutive tissue sections and this will be particularly important for examining branched anatomical structures such as breast, prostate and lung.

References

1. Bankhead, P. *et al.* QuPath: Open source software for digital pathology image analysis. *Sci. Rep.* **7**, 16878 (2017).
2. Kozarewa, I. *et al.* Amplification-free Illumina sequencing-library preparation facilitates improved mapping and assembly of (G+C)-biased genomes. *Nat. Methods* **6**, 291–295 (2009).
3. Li, H. & Durbin, R. Fast and accurate short read alignment with Burrows-Wheeler transform. *Bioinformatics* **25**, 1754–1760 (2009).
4. Yates, L. R. *et al.* Genomic Evolution of Breast Cancer Metastasis and Relapse. *Cancer Cell* **32**, 169–184.e7 (2017).
5. Yates, L. R. *et al.* Subclonal diversification of primary breast cancer revealed by multiregion sequencing. *Nat. Med.* **21**, 751–759 (2015).
6. Dentro, S. C., Wedge, D. C. & Van Loo, P. Principles of Reconstructing the Subclonal Architecture of Cancers. *Cold Spring Harb. Perspect. Med.* **7**, (2017).
7. Nik-Zainal, S. *et al.* The life history of 21 breast cancers. *Cell* **149**, 994–1007 (2012).
8. Bolli, N. *et al.* Heterogeneity of genomic evolution and mutational profiles in multiple myeloma. *Nat. Commun.* **5**, 2997 (2014).
9. Svedlund, J. *et al.* Generation of in situ sequencing based OncoMaps to spatially resolve gene expression profiles of diagnostic and prognostic markers in breast cancer. *EBioMedicine* **48**, 212–223 (2019).
10. Ke, R. *et al.* In situ sequencing for RNA analysis in preserved tissue and cells. *Nat. Methods* **10**, 857–860 (2013).
11. Hörl, D. *et al.* BigStitcher: reconstructing high-resolution image datasets of cleared and expanded samples. *Nat. Methods* **16**, 870–874 (2019).

12. Bradski, G. The openCV library. *Dr. Dobb's Journal: Software Tools for the Professional Programmer* **25**, 120–123 (2000).

13. van der Walt, S. *et al.* scikit-image: image processing in Python. *PeerJ* **2**, e453 (2014).

14. Arganda-Carreras, I., Sorzano, C., Kybic, J. & Ortiz-de-Solórzano, C. bUnwarpJ : Consistent and Elastic Registration in ImageJ . Methods and Applications. (2008).

15. Gataric, M. *et al.* PoSTcode: Probabilistic image-based spatial transcriptomics decoder. *bioRxiv* 2021.10.12.464086 (2021) doi:10.1101/2021.10.12.464086.

16. Marcon, E. & Puech, F. Measures of the geographic concentration of industries: improving distance-based methods. *J. Econ. Geogr.* **10**, 745–762 (2010).

17. Marcon, E., Traissac, S., Puech, F. & Lang, G. Tools to Characterize Point Patterns: dbmss for R. *J. Stat. Softw.* **67**, 1–15 (2015).

18. Caicedo, J. C. *et al.* Nucleus segmentation across imaging experiments: the 2018 Data Science Bowl. *Nat. Methods* **16**, 1247–1253 (2019).

19. Wu, S. Z. *et al.* A single-cell and spatially resolved atlas of human breast cancers. *Nat. Genet.* **53**, 1334–1347 (2021).

20. Ruifrok, A. C. & Johnston, D. A. Quantification of histochemical staining by color deconvolution. *Anal. Quant. Cytol. Histol.* **23**, 291–299 (2001).

21. Salvatier, J., Wiecki, T. V. & Fonnesbeck, C. Probabilistic programming in Python using PyMC3. *PeerJ Comput. Sci.* **2**, e55 (2016).

22. Handsaker, R. E. *et al.* Large multiallelic copy number variations in humans. *Nat. Genet.* **47**, 296–303 (2015).

23. Shao, X. *et al.* Copy number variation is highly correlated with differential gene expression: a pan-cancer study. *BMC Med. Genet.* **20**, 175 (2019).

24. Kucukelbir, A., Tran, D., Ranganath, R., Gelman, A. & Blei, D. M. Automatic Differentiation Variational Inference. *arXiv [stat.ML]* (2016).

25. Phan, D., Pradhan, N. & Jankowiak, M. Composable Effects for Flexible and Accelerated Probabilistic Programming in NumPyro. *arXiv [stat.ML]* (2019).

26. Ellis, P. *et al.* Reliable detection of somatic mutations in solid tissues by laser-capture

microdissection and low-input DNA sequencing. *Nat. Protoc.* **16**, 841–871 (2021).

27. Nik-Zainal, S. *et al.* Landscape of somatic mutations in 560 breast cancer whole-genome sequences. *Nature* **534**, 47–54 (2016).
28. van Luijt, P. A. *et al.* The distribution of ductal carcinoma in situ (DCIS) grade in 4232 women and its impact on overdiagnosis in breast cancer screening. *Breast Cancer Res.* **18**, 47 (2016).
29. Gupta, S. K., Douglas-Jones, A. G., Fenn, N., Morgan, J. M. & Mansel, R. E. The clinical behavior of breast carcinoma is probably determined at the preinvasive stage (ductal carcinoma in situ). *Cancer* **80**, 1740–1745 (1997).
30. Rakha, E., Toss, M. & Quinn, C. Specific cell differentiation in breast cancer: a basis for histological classification. *J. Clin. Pathol.* **75**, 76–84 (2022).
31. Nirmal, A. J. *et al.* The spatial landscape of progression and immunoediting in primary melanoma at single cell resolutionAtlas of Primary Melanoma. *Cancer Discov.* (2022).
32. Janiszewska, M. *et al.* Subclonal cooperation drives metastasis by modulating local and systemic immune microenvironments. *Nat. Cell Biol.* **21**, 879–888 (2019).
33. Turajlic, S., Sottoriva, A., Graham, T. & Swanton, C. Resolving genetic heterogeneity in cancer. *Nat. Rev. Genet.* **20**, 404–416 (2019).
34. Lieberman, E., Hauert, C. & Nowak, M. A. Evolutionary dynamics on graphs. *Nature* **433**, 312–316 (2005).
35. Noble, R. *et al.* Spatial structure governs the mode of tumour evolution. *Nat Ecol Evol* **6**, 207–217 (2022).
36. West, J., Schenck, R. O., Gatenbee, C., Robertson-Tessi, M. & Anderson, A. R. A. Normal tissue architecture determines the evolutionary course of cancer. *Nat. Commun.* **12**, 2060 (2021).
37. Sharonov, G. V., Serebrovskaya, E. O., Yuzhakova, D. V., Britanova, O. V. & Chudakov, D. M. B cells, plasma cells and antibody repertoires in the tumour microenvironment. *Nat. Rev. Immunol.* **20**, 294–307 (2020).
38. Vickovic, S. *et al.* High-definition spatial transcriptomics for in situ tissue profiling. *Nat. Methods* **16**, 987–990 (2019).
