# Supplementary material for: Spatial genomics maps the structure, nature and evolution of cancer clones
Source: Nature. 2022 Nov 9;611(7936):594–602. doi: 10.1038/s41586-022-05425-2 (PMC9668746; doi:10.1038/s41586-022-05425-2)
Supplement: Supplementary file 2 — Reporting Summary [file 41586_2022_5425_MOESM2_ESM.pdf]

nature portfolio

Corresponding author(s): Lucy R Yates, Mats Nilsson, Moritz Gerstung

Last updated by author(s): 31/08/22

Reporting Summary

Nature Portfolio wishes to improve the reproducibility of the work that we publish. This form provides structure for consistency and transparency in reporting. For further information on Nature Portfolio policies, see our Editorial Policies and the Editorial Policy Checklist.

Statistics

For all statistical analyses, confirm that the following items are present in the figure legend, table legend, main text, or Methods section.

n/a	Confirmed	
☐	☒	The exact sample size (*n*) for each experimental group/condition, given as a discrete number and unit of measurement
☐	☒	A statement on whether measurements were taken from distinct samples or whether the same sample was measured repeatedly
☐	☒	The statistical test(s) used AND whether they are one- or two-sided<br>*Only common tests should be described solely by name; describe more complex techniques in the Methods section.*
☐	☒	A description of all covariates tested
☐	☒	A description of any assumptions or corrections, such as tests of normality and adjustment for multiple comparisons
☐	☒	A full description of the statistical parameters including central tendency (e.g. means) or other basic estimates (e.g. regression coefficient) AND variation (e.g. standard deviation) or associated estimates of uncertainty (e.g. confidence intervals)
☐	☒	For null hypothesis testing, the test statistic (e.g. *F*, *t*, *r*) with confidence intervals, effect sizes, degrees of freedom and *P* value noted<br>*Give P values as exact values whenever suitable.*
☐	☒	For Bayesian analysis, information on the choice of priors and Markov chain Monte Carlo settings
☒	☐	For hierarchical and complex designs, identification of the appropriate level for tests and full reporting of outcomes
☐	☒	Estimates of effect sizes (e.g. Cohen's *d*, Pearson's *r*), indicating how they were calculated

Our web collection on statistics for biologists contains articles on many of the points above.

Software and code

Policy information about availability of computer code

Data collection

Subclonal clusters were acquired as reported in previous publications using DPClust https://github.com/Wedge-Oxford/dpclust Version 2.2.8
QuPath software (Version 0.3.0) https://qupath.github.io/

Large scale probe design was facilitated using an in-house Python (3) software package as described previously (Ref PMID 31740815) which utilizes ClustalW and BLAST+ to ensure probe specificity.

From the total of 51 image sets, 43 were stitched with Carl-Zeiss ZEN software (version 3.1), and the other 8 failed image sets were stitched using BigStitcher (version 0.9). The registration across imaging cycles was performed in two steps: affine registration on DAPI channel and subsequently local warping on anchor channel. For both steps we used algorithms provided in libraries OpenCV-contrib (version 4.3.0) and scikit-image (version 0.17).

Analysis of sample P2-LN required IHC signal projection performed on a consecutive slide back to BaSISS slide. To achieve this, we performed a spline-based elastic registration implemented in ImageJ package UnwarpJ(Arganda-Carreras et al. 2008).

Nuclei segmentation code is available at yozhikoff/segmentation

Data analysis

The manuscript used publicly available, open source R and Python libraries/packages for data analysis as described in the methods section. All scripts and custom code for data analysis, including step-by-step notebooks are available at GitHub repository (https://github.com/gerstung-lab/BaSISS) and under the DOI: 10.5281/zenodo.703731

Software and package version used during the analysis:

Python (3.8.12) with packages:
- numpy (1.22.2)
- pandas (1.1.5)
- scipy (1.7.3)
- opencv (4.5.5)
- matplotlib (3.5.1)
- pymc (4.0.1)
- numpyro (0.10.0)
- scikit-image (0.19.1)
- scikit-learn (1.0.2)
- scanpy (1.8.1)
- shapely (1.8.0)
R (4.1.3) with packages:
- dbmss (2.7-10)

For manuscripts utilizing custom algorithms or software that are central to the research but not yet described in published literature, software must be made available to editors and reviewers. We strongly encourage code deposition in a community repository (e.g. GitHub). See the Nature Portfolio guidelines for submitting code & software for further information.

Data

Policy information about availability of data

All manuscripts must include a data availability statement. This statement should provide the following information, where applicable:

- Accession codes, unique identifiers, or web links for publicly available datasets
- A description of any restrictions on data availability
- For clinical datasets or third party data, please ensure that the statement adheres to our policy

All figures are derived from data that is available for download via ftp://ftp.sanger.ac.uk/pub/cancer/LomakinEtAl_BaSISS
Bulk tissue whole genome sequencing data are deposited in the European Genome Phenome Archive and are available for download on request (EGA, https://ega-archive.org/datasets) with the following accessions: EGAD00001002696 (P2 samples: with IDs PD14780a,b,d,e) and EGAD00001000898 (P1 samples: with IDs PD9694a,b,c,d).

Registered fluorescent microscopy images from in-situ sequencing experiments are deposited at BioImage Archive under the accession number S-BIAD537.

Public data used for single cell RNA-seq analysis were obtained from the NCBI's Gene Expression Omnibus (https://www.ncbi.nlm.nih.gov/geo/query/acc.cgi?acc=GSE176078)

Human research participants

Policy information about studies involving human research participants and Sex and Gender in Research.

Reporting on sex and gender	Both participants reported female sex, gender is not relevant
Population characteristics	Participants are female, age 37 and 66 years old at time of diagnosis and surgery for multifocal breast cancers of no special type. No prior cancer diagnosis or treatment was administered. No genotyping was performed on patients.
Recruitment	At the time of tissue collection all patients with a diagnosis of primary breast cancer, attending Dana-Farber Cancer Institute were invited to participate in project SHARE. Participants provided written consent for inclusion in the study that entails the donation of tissue from planned clinical procedures (that exceeds pathological diagnostic requirements and would normally be discarded) and clinical data for research purposes. The specific pathological specimens in this analysis were identified by the local pathologist based on the presence of additional histological stages of disease (an involved lymph node or extensive pre-cancerous lesion) and the availability of sufficient tissue blocks to perform the planned experiments. It is feasible that the amount of intra-tumour heterogeneity observed in this study exceeds what would be observed in some smaller primary breast cancers without multiple histological features.
Ethics oversight	Samples and data were obtained and managed in line with the declaration of Helsinki under "project SHARE" #93-085, approved by the Dana-Farber Harvard Cancer Center Institutional Review Board. Sample and data handling at the Wellcome Sanger Institute, Cambridgeshire, UK was performed under the wider framework and approval for the Breast Cancer Genome Analyses for the International Cancer Genome Consortium Working Group under REC reference: 09/H0306/36 (Cambridgeshire 3 Research Ethics Committee). The study was later transferred to a protocol REC: 20/PR/0905 (London-Harrow Research Ethics Committee).

Note that full information on the approval of the study protocol must also be provided in the manuscript.

Field-specific reporting

Please select the one below that is the best fit for your research. If you are not sure, read the appropriate sections before making your selection.

☒ Life sciences ☐ Behavioural & social sciences ☐ Ecological, evolutionary & environmental sciences

For a reference copy of the document with all sections, see nature.com/documents/nr-reporting-summary-flat.pdf

Life sciences study design

All studies must disclose on these points even when the disclosure is negative.

Sample size	This was a biological study, not a clinical trial so we did not undertake a power calculation for the number of patients. A total of 8 tissue samples from 2 donors was considered sufficient to develop and demonstrate the spatial profiling techniques.
Data exclusions	All patients and samples analysed were included in the data presented. No samples were excluded.
Replication	For samples P1-ER1, P1-ER2, P1-D1 , P2-TN2 a replicate BaSISS experiment was conducted with a slightly altered ISS protocol. Phi29 buffer (Thermo Fisher 10X reaction buffer: 330 mM Tris-acetate (pH 7.9 at 37°C), 100 mM Mg-acetate, 660 mM K-acetate, 1% Tween 20 and 10 mM DTT) and no Exonuclease 1 in the rolling circle amplification step. Results of clone mapping are shown in Extended Data Fig. 5 - clone field distributions are largely replicated.<br>For P1-ER1 and P1-ER2 whole genome sequencing of laser capture microdissected regions that were selected based upon an ability to identify them in both LCM and BaSISS z-stack sections. All regions that fulfilled this criteria validated the BaSISS model and are shown in Fig.2. For some regions including those in P1-D1 and P1-D2 the tissue structure was such that we could not link regions in the z-plane. Nonetheless, although regional clone compositions could not be validated, the general patterns of mutation/clone co-occurrence and segregation could be confirmed from these data. Tissue availability precluded LCM in patient P2.
Randomization	This was a biological study and not a clinical trial and therefore we did not randomise subjects.
Blinding	This was a biological study and not a clinical trial and therefore we did not blind. Histopathological annotation was performed by qualified clinical pathologists without prior knowledge of the spatial genomic data.

Reporting for specific materials, systems and methods

We require information from authors about some types of materials, experimental systems and methods used in many studies. Here, indicate whether each material, system or method listed is relevant to your study. If you are not sure if a list item applies to your research, read the appropriate section before selecting a response.

Materials & experimental systems

n/a	Involved in the study	
☐	☒	Antibodies
☒	☐	Eukaryotic cell lines
☒	☐	Palaeontology and archaeology
☒	☐	Animals and other organisms
☒	☐	Clinical data
☒	☐	Dual use research of concern

Methods

n/a	Involved in the study	
☒	☐	ChIP-seq
☒	☐	Flow cytometry
☒	☐	MRI-based neuroimaging

Antibodies

Antibodies used	panCK, Dako(Agilent), 1:100, Catalogue number:M3515, clone AE1/AE3, LOT: L11139890<br>CD45, 1:100, Dako(Agilent), Catalogue number:M0701, clone 2B11 + PD7126, LOT:20026786<br>Her2/ErbB2, 1:50, CellSignaling, Catalogue number:4290T, clone:D8Fl2, LOT:2<br>SM-MHC, 1:100, BioCare Medical LLC, Catalogue number:CM 420B, mouse monoclonal, clone: SMMS-1, LOT number unknown<br>P63, 1:150, BioCare Medical LLC, Catalogue number:CM 163C, mouse monoclonal, clone: BC4A4, LOT number unknown<br>PR, DakoCytomation, 1:75, Catalogue number:M3569, mouse monoclonal, clone:PgR636, LOT number unknown<br>Ki67, DakoCytomation, 1:400, Catalogue number:M7240, mouse monoclonal, clone: MIB-1, LOT number unknown<br>PTEN, Abcam Anti-PTEN antibody, 1:500, Catalogue number:ab267787, clone:EPR22636-122, LOT number unknown<br>PTEN, Santa Cruz Biotechnologies, 1:300, Catalogue number:sc-7974, clone:A2B1, LOT number unknown<br>PTEN Cell Signalling, 1:400, Catalogue number:9559, clone:138G6, LOT number unknown<br>PTEN Cell Signalling, 1:500, Catalogue number:9188, clone:D4.3, LOT number unknown<br>ImmPRESS HRP Anti-mouse IgG, VectorLaboratories, Catalogue number:MP-7402-50 ready-to-use, clone not applicable, LOT number unknown<br>ImmPRESS HRP Anti-rabbit IgG, VectorLaboratories, Catalogue number:MP-7401-50, ready-to-use, clone not applicable, LOT number unknown<br>Labelled polymer-HRP anti-mouse (polymer-M): DakoCytomation, Catalogue number: K4007<br>Poly-AP anti-mouse IgG (poly-AP-M): Leica, Catalogue number: PV6110
Validation	panCK - Approved for in vitro diagnistics by IHC (CE-IVD) according to datasheet. No specific IHC validation reported by manufacturer. Antibody AEI immunoreacts with an antigenic determinant present on most of the subfamily A cytokeratins, including cytokeratins 10, 13, 14, 15 16 and 19. Antibody AE3 reacts with an antigenic determinant shared by the subfamily B cytokeratins including 1, 2, 3, 4, 5, 6, 7 and 8. Please see suppliers datasheet for corresponding references.

CD45 - Approved for in vitro diagnistics by IHC (CE-IVD) according to datasheet.
Her2/ErbB2 - Recommended for IHC by the supplier; according to datasheet this antibody may cross-react slightly with other overexpressed RTKs. No specific IHC validation reported by manufacturer.

For Her2: no IHC validation reported by the manufacturer; the manufacturer demonstrates that the antibody recognizes a protein band at 185kDa in Western Blot analysis of lysates from Her2 positive human cells (MCF7 and SKBR3).

PTEN IHC was validated using several anti-PTEN antibodies (Cell Signaling Technologies: anti-PTEN (D4.3) Antibody (dilution 1:50) and anti-PTEN (138G6) Antibody; Santa Cruz Biotechnologies anti-PTEN Antibody (A2B1) (dilution 1:300); and Abcam anti-PTEN Antibody [EPR22636-122], dilution 1:500). The Abcam anti-PTEN Antibody [EPR22636-122] displayed the best signal on frozen tissue sections and was used in the study.

SMMHC, P63, Ki-67 and PR antibodies are used by the clinical service at Brigham and Women's Hospital and undergo validation as part of clinical use in Clinical Laboratory Improvement Amendments (CLIA) certified pathology laboratory using control samples (e.g., breast carcinoma, tonsil, etc) as recommended by the suppliers.
